# Classification of Fibrillation Organisation Using Electrocardiograms to Guide Mechanism-Directed Treatments

**DOI:** 10.3389/fphys.2021.712454

**Published:** 2021-11-11

**Authors:** Xinyang Li, Xili Shi, Balvinder S. Handa, Arunashis Sau, Bowen Zhang, Norman A. Qureshi, Zachary I. Whinnett, Nick W. F. Linton, Phang Boon Lim, Prapa Kanagaratnam, Nicholas S. Peters, Fu Siong Ng

**Affiliations:** ^1^National Heart and Lung Institute, Imperial College London, London, United Kingdom; ^2^Department of Bioengineering, Imperial College London, London, United Kingdom

**Keywords:** fibrillation, cardiac arrhythmia, electrocardiography, electrograms, ablation

## Abstract

**Background:** Atrial fibrillation (AF) and ventricular fibrillation (VF) are complex heart rhythm disorders and may be sustained by distinct electrophysiological mechanisms. Disorganised self-perpetuating multiple-wavelets and organised rotational drivers (RDs) localising to specific areas are both possible mechanisms by which fibrillation is sustained. Determining the underlying mechanisms of fibrillation may be helpful in tailoring treatment strategies. We investigated whether global fibrillation organisation, a surrogate for fibrillation mechanism, can be determined from electrocardiograms (ECGs) using band-power (BP) feature analysis and machine learning.

**Methods:** In this study, we proposed a novel ECG classification framework to differentiate fibrillation organisation levels. BP features were derived from surface ECGs and fed to a linear discriminant analysis classifier to predict fibrillation organisation level. Two datasets, single-channel ECGs of rat VF (*n* = 9) and 12-lead ECGs of human AF (*n* = 17), were used for model evaluation in a leave-one-out (LOO) manner.

**Results:** The proposed method correctly predicted the organisation level from rat VF ECG with the sensitivity of 75%, specificity of 80%, and accuracy of 78%, and from clinical AF ECG with the sensitivity of 80%, specificity of 92%, and accuracy of 88%.

**Conclusion:** Our proposed method can distinguish between AF/VF of different global organisation levels non-invasively from the ECG alone. This may aid in patient selection and guiding mechanism-directed tailored treatment strategies.

## 1. Introduction

Atrial fibrillation (AF) and ventricular fibrillation (VF) are complex heart rhythm disorders with an increasing prevalence (Zheng et al., 2001; Morillo et al., 2017; Martín-Yebra et al., 2019). Both AF and VF show beat-to-beat variability in electrical propagation through the myocardium and the mechanisms that initiate and sustain these rhythms are not entirely understood.

The limited insight into mechanisms of myocardial fibrillation stems primarily from *ex vivo* optical mapping studies of the transmembrane potentials with potentiometric dyes (Laughner et al., 2012), which have shown several competing mechanisms (Handa et al., 2021). The multiple wavelet hypothesis proposes that fibrillation is a chaotic disorganised rhythm sustained by multiple wavelets of electrical activity that meander, collide, and continuously regenerate (Moe et al., 1964; Krummen et al., 2016). The competing hypothesis is that fibrillation is a spatiotemporally organised phenomenon sustained by one or more rotational drivers (RDs). RDs are scroll waves of electrical propagation that perpetuate around a point of phase singularity, that can anchor to specific regions and/or meander through the myocardium, generating fibrillation wavefronts (Pandit and Jalife, 2013). Multiple disorganised rapidly discharging foci within the myocardium have also been shown to sustain fibrillation (Lee et al., 2015), while, more recently, a more complex mechanism of asynchronous endo-epicardial disassociation of fibrillatory conduction has been proposed in AF (de Groot et al., 2016).

Treatment options for patients at risk of VF and those suffering from AF are empirical at present and not targeted towards the specific mechanism of fibrillation. VF survivors who are at further risk of future episodes are conventionally offered implantable cardioverter defibrillation to terminate VF episodes, while pulmonary vein isolation (PVI) to electrically disconnect the atrial body from the pulmonary veins (where rapid firing can trigger AF) is the only proven efficacious treatment in AF (Sau et al., 2019). The absence of any mechanism-directed treatment for patients with AF in particular has led to limited success rates in catheter ablation for persistent AF (Schreiber et al., 2015). There is a pressing need to move beyond the one-size-fits-all approach of empirical treatment towards mechanism-directed treatments.

We recently showed that there is a range of AF and VF mechanisms, with varying degrees of the global organisation, using *ex vivo* optical mapping of explanted perfused hearts and invasive intracardiac mapping in patients undergoing AF ablation (Handa et al., 2020). Only some forms of AF are globally organised and driven by stable RDs, and these would be potentially amenable to ablation targeting RDs, while other forms of AF are globally disorganised with no clear drivers and as such may respond to compartmentalisation of the atria. A possible approach to individualised tailored therapy would be to select the appropriate treatments based on the specific electrophysiological mechanisms sustaining fibrillation in each specific patient (Ng et al., 2020). Ideally, we would be able to identify the mechanism non-invasively.

The electrocardiogram (ECG) is an integral part of cardiac diagnostics and routine care. With the advent of machine learning, there has been increasing interest in extending the diagnostic abilities of ECGs beyond qualitative human assessment (Fan et al., 2018). Signal processing of ECGs has been implemented in AF (Meo et al., 2013), where certain features of ECG complexity have been shown to correlate with the long-term success of catheter ablation (Lankveld et al., 2016). Conventional signal processing techniques, in the form of dominant frequency (DF) analysis (Uetake et al., 2014) and entropy analysis (Alcaraz and Rieta, 2012) have been utilised to analyse AF surface ECGs, in addition to more novel techniques such as fibrillation-wave power (FWP) and fibrillation-wave amplitude (FWA) analysis (Lankveld et al., 2016) to predict treatment outcomes. A recent study employed convolutional neural networks to identify the ECG signatures of AF from normal sinus rhythm ECGs alone with an accuracy of 83.3% and proposed it as a tool to eliminate the need for expensive long-duration ECG monitoring to diagnose AF (Attia et al., 2019).

In this study, we sought to investigate whether the degree of the global organisation of VF and AF and the underlying fibrillation mechanisms itself can be detected with a machine learning classification framework based on the non-invasive ECG recording alone. First, epicardial VF activity was recorded in *ex vivo* explanted perfused rat hearts with high spatial resolution optical mapping. The mechanism was classified using phase analysis as either globally chaotic and driven by multiple wavelets, or globally organised and driven by RDs. This characterisation was designated as ground truth for labelling the corresponding single channel ECG recorded during the optical mapping studies and to train a machine learning model (Li et al., 2019; Handa et al., 2020). After developing and validating our characterisation of VF mechanisms from ECGs, we trained the model on human AF surface ECGs in patients with persistent AF to determine the prediction accuracy in the classification of the underlying AF organisation/mechanism as determined by invasive intracardiac mapping. For both data sets, the proposed classification frameworks were evaluated in a leave-one-out (LOO) manner, and classification results showed that the proposed method correctly predicted organisation level from rat VF ECGs sensitivity of 75%, specificity of 80%, and accuracy of 78%, and from clinical AF ECG with the sensitivity of 80%, specificity of 92%, and accuracy of 88%. Accurate classification of fibrillation organisation and mechanism using the ECG may allow for more tailored treatments based on the specific arrhythmia mechanism.

## 2. Data Acquisition and Data Labelling

The objective of the proposed classification framework is to differentiate between organised and disorganised forms of fibrillation from surface ECG. Organised fibrillation is usually driven by RDs while disorganised fibrillation is by multiple wavelets (Handa et al., 2020). This novel technique could be ultimately used to guide patient selection for individualised treatment options. The two data sets used for the model evaluation were derived from a recent study by our group, and the methodology for data acquisition has previously been described in detail (Handa et al., 2020). Optical mapping VF data were obtained by performing *ex vivo* perfused rat heart optical mapping of the transmembrane potential and the clinical AF data were acquired from patients in persistent AF using intracardiac multipolar catheter recordings of electrograms (EGMs) during catheter ablation procedures. Concurrent surface ECGs were also recorded for rat VF and human AF.

**Figure 1:**
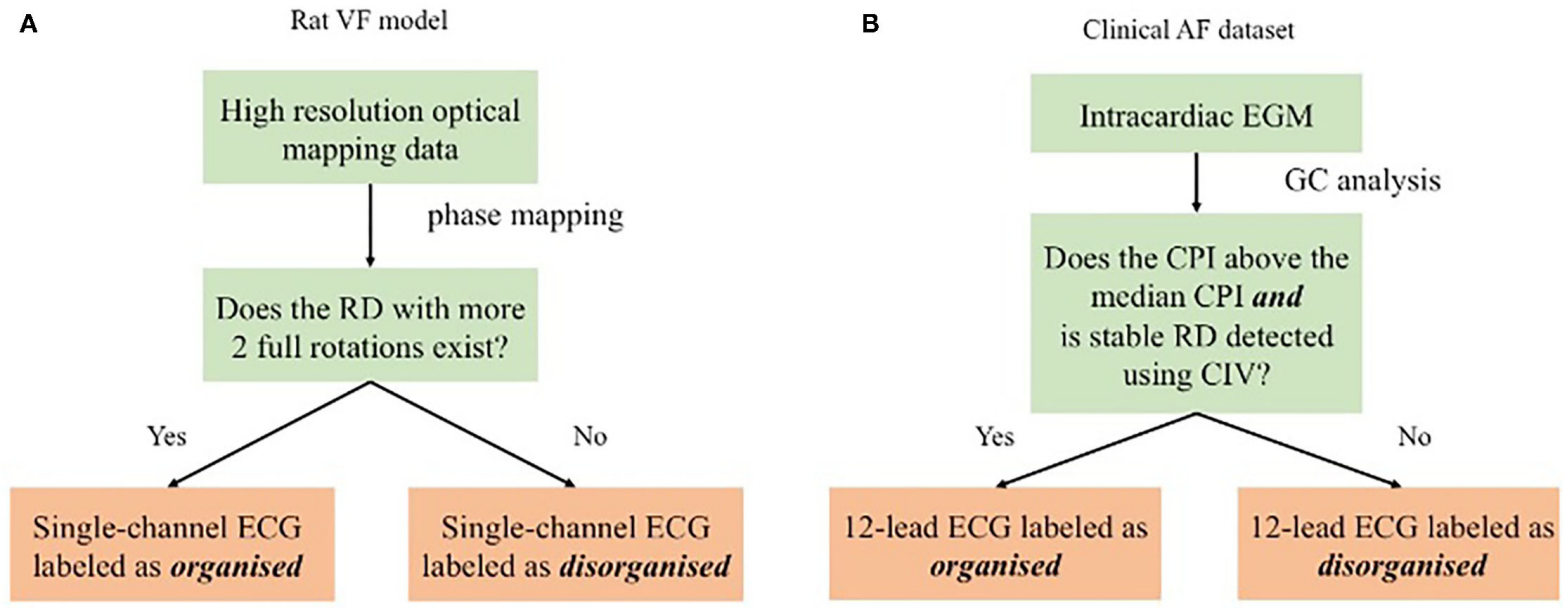
Schematic showing the proposed method evaluated by different sets. For the rat ventricular fibrillation (VF) data, as shown in **(A)**, the ECG was labelled by the rotational driver (RD) detection results from phase mapping. For the clinical atrial fibrillation (AF) data, as shown in **(B)**, GC analysis of the electrogram (EGM) data was the surrogate organisation label for the ECG classification.

For the rat VF model, the labelling of the fibrillation organisation level was conducted using phase mapping, described below. For the clinical AF data, where the high-resolution recording was not available, a Granger causality (GC) analysis of the intracardiac EGM data was used for labelling the clinical ECG. A schematic of the study design is shown in Figure 1. The methodology for phase analysis and GC analysis has been described in detail (Handa et al., 2020). Details of the analysis techniques and labelling are presented briefly in the following section.

Data for the AF mapping studies were collected in the cardiac electrophysiology lab, Hammersmith Hospital. Approval was given by the Local Research and Ethics Committee for Imperial College Healthcare NHS Trust and written informed consent was obtained from all patients. LA mapping data were obtained for 17 patients with persistent AF. Electroanatomical mapping data were collected using the EnSite™ Velocity™ system (Abbott Inc, Minnesota, USA). On the day of the procedure, all patients were presented in AF. Left atrial access was gained with a transeptal puncture. A 20-ring electrode A-Focus II™ (Abbot Inc., Minnesota, USA) mapping catheter (double loop, 1 mm length electrodes, 4 mm interelectrode spacing) was used to acquire LA geometry and EGM. EGM were collected with stable tissue contact at the endocardial surface. Data from pulmonary veins and left atrial appendage were excluded from the analysis. Data were collected in both persistent AF and a subset of patients in sinus rhythm after direct current cardioversion. The bipolar EGMs were filtered at 30–500 Hz bandpass filtering. The endocardial area subtended by the A-FocusII™ mapping catheter was termed a 'kernel'. For each given kernel, 20 s of data were subsequently analysed.

The number of kernels collected and subsequently the number of segments analysed in this paper varied between patients due to differing left atrial geometry and catheter stability. An attempt was made to map the left atrium extensively for all subjects, thus, the impact of the heterogeneity will be minimised in the final analysis. A summary of patient characteristics can be found in Table 1.

**Table 1:** Patient characteristics of invasive clinical data-set.

**Patient characteristics (*****n*** **= 17)**	
Age (years)	66 ± 7
Male	10
Mean left atrium size on TTE (mm)	44 ± 5
Mean CHA_2_DS_2_VASc score	2.5(0-6)
Hypertension	7
Diabetes Mellitus	4
Cerebrovascular Disease	2
History of heart failure	3
Duration of persistent AF (months)	20.5 ± 9

### 2.1. Phase Mapping of Rat VF Data

For the rat VF model, nine *ex vivo* perfused rat hearts underwent high-resolution optical mapping of the left ventricular epicardial surface after VF induction with programmed electrical stimulation, and the single-channel ECG was recorded simultaneously, with a sampling rate of 1,000 Hz.

Phase analysis is a gold standard technique for the analysis of fibrillatory signals (Nattel et al., 2017). Phase mapping was applied to the optical mapping data to identify phase singularities (PS) and RDs for further labelling. All our methods for analysing optical mapping fluorescence data have been previously described in detail (Ng et al., 2013, 2016; Roney et al., 2017; Li et al., 2019; Handa et al., 2020). In this study, a RD was defined as a PS with more than two full rotations. Figure 1A shows the ECG labelling based on the RD identification: ECGs are identified as organised if spatiotemporally stable RDs sustained VF on optical mapping and disorganised if VF was driven by chaotic wavefronts with no identifiable stable RD.

### 2.2. GC Analysis of Clinical AF Data

For clinical AF data (17 subjects), alternative analysis techniques based on GC analysis were used to classify organisational levels of fibrillation recordings as phase analysis of intracardiac EGMs is confounded by several issues, including the low spatial resolution of clinical data (Roney et al., 2017).

Granger causality is a measurement of signal inter-dependency and has been previously used to delineate dominant patterns of wavefront propagation in fibrillation (Luengo et al., 2016, 2018; Rodrigo et al., 2016; Alcaine et al., 2017). In our previous study, two measurements derived from GC, causality pairing index (CPI) and circular interdependence value (CIV) were applied to intra-cardiac EGM to quantify the AF organisation level, detect RDs, and identify the likely mechanism sustaining fibrillation (Handa et al., 2020). In this study, the CPI and CIV calculated from intra-cardiac electrogram data (EGM) were used to label the corresponding surface ECGs as organised or disorganised fibrillation. To make this work self-contained, the calculation of CPI and CIV will be presented in the following section.

#### 2.2.1. Causality Pairing Index

From the multi-variate cardiac signal, $\text{x}\left(t\right)\in R^{n_{c}}$ at time *t* of the dimension of *n*_*c*_, GC is inferred by fitting an auto-regressive (AR) model to **x**(*t*) as


(1) $$\begin{array}{r} Â\left(\tau \right)=\arg\underset{A\left(\tau \right)}{\min}\overset{n_{t}}{\underset{t=L+1}{\sum }}||\text{x}\left(t\right)-\overset{L}{\underset{\tau =1}{\sum }}A{\left(\tau \right)}^{⊤}\text{x}\left(t-\tau \right)|{|}_{2}+\\ \;\lambda \overset{L}{\underset{\tau =1}{\sum }}||A\left(\tau \right)|{|}_{1},\;\tau =1,\ldots ,L \end{array}$$

$A\left(\tau \right)\in R^{n_{c}\times n_{c}}$ is the AR coefficient matrix, τ is the time lag, *L* is the maximal time lag of the model, and λ is a regularisation coefficient. Let *x*_*i*_(*t*) be the *i*-th row of **x**(*t*). The element of the *i*-th row and *j*-th column, *A*(τ, *i, j*), reflects the strength of the *x*_*i*_(*t*−τ) in predicting *x*_*j*_(*t*), or in other words, the temporal dependency between *x*_*i*_(*t*) and *x*_*j*_(*t*).

The optimisation problem in Equation 1 is usually termed as the Lasso-Granger approach (Valdés-Sosa et al., 2005; Arnold et al., 2007; Song and Bickel, 2011). With the *l*_1_-norm-based regularisation term $\overset{L}{\underset{\tau =1}{\sum }}||A\left(\tau \right)|{|}_{1}$, the Lasso-Granger approach yields a more sparse and robust Granger causality estimation. In this study, Forward Backward Lasso Granger Causality is applied to solve (Equation 1), which is faster and more robust (Cheng et al., 2014).

With the *l*_1_-norm sparsity constraint, solving (Equation 1) drives all elements in Â(τ) to be zero unless the casual relationships between certain pairs of signals are very strong. Therefore, a measurement of the organisation was calculated as the percentage of the non-zero pairings between different signals.

To be specific, define S as the set containing all the non-zero elements in Â(τ), i.e.,


(2) $$\begin{array}{r} S=\{â\left(\tau ,i,j\right)\;|a\left(\tau ,i,j\right)>0,i\neq j,\\ \tau =1,\ldots ,L,\;\text{and}i,j=1,\ldots .,n_{c}\} \end{array}$$

where â(τ, *i, j*) is the element of *i*-th row and *j*-th column in Â(τ). The CPI is obtained as the following by


(3) $$\begin{array}{l} CPI=\frac{\left|S\right|}{L\left(n_{c}^{2}-n_{c}\right)} \end{array}$$

where |·| is the cardinal number of a set.

By Equation (3), CPI quantifies the global fibrillatory organisation by calculating the number of possible Granger-causal signal pairs in fibrillation between which there are propagational effects on a normalised scale of 0–1, where 0 is defined as no possible pairing having causal dependency (most disorganised) and 1 where all possible pairings have causal dependency (most disorganised).

#### 2.2.2. Circular Interdependence Value

Circular interdependence value is an analytical tool for localising RDs from regional analysis of cardiac signals from the flow directions indicated by Â(τ). Let **x**_*i*_(*t*) be the *i*-th signal of **x**(*t*). The major source index *s*_*i*_ for **x**_*i*_(*t*) is defined as the signal with the strongest causal influence on **x**_*i*_(*t*), i.e.,


(4) $$\begin{array}{l} s_{i}=\arg\underset{j}{\max}\underset{\tau }{\sum }Â\left(\tau ,i,j\right),\;j=1,\ldots ,n_{c}\;\text{and}j\neq i \end{array}$$

Let **p**_*s*_*i*__ and **p**_*i*_ be the coordinates of the locations corresponding to **x**_*i*_(*t*) and **x**_*s*_*i*__(*t*) in a global coordinate, respectively, and the GC vector for **x**_*i*_(*t*), **g**_*i*_, is calculated as the following


(5) $$\begin{array}{l} {\text{g}}_{i}=\{\begin{array}{cc} {\text{p}}_{s_{i}}-{\text{p}}_{i} & \text{if}\sum_{\tau }Â\left(\tau ,i,s_{i}\right)>0.\\ 0 & \text{otherwise}. \end{array} \end{array}$$

The GC vector **g**_*i*_ in Equation (5) can be regarded as the source-to-sink vector for electrode *i*, pointing from its source electrode *s*_*i*_ to electrode *i*. Let **p**_0_ be the coordinates of the location of interest. Then, then rotational direction for **p**_*i*_ relative to **p**_0_ could be calculated as the cross product of **p**_*i*_ − **p**_0_ and **g**_*i*_ after normalisation, i.e.,


(6) $$\begin{array}{l} {\text{r}}_{i}=\frac{{\text{p}}_{i}-{\text{p}}_{0}}{\left||{\text{p}}_{i}-{\text{p}}_{0}|\right|}\times \frac{{\text{g}}_{i}}{\left||{\text{g}}_{i}|\right|}\\ \;\equiv r_{i,1}i+r_{i,2}j+r_{i,3}k \end{array}$$

where ***i***, ***j***, and ***k*** are the standard basis vectors corresponding to the x-, y-, and z-axis in the global coordinate, respectively. Suppose a local coordinate where the x-y plane is specified by **p**_0_, **p**_*i*_, and **p**_*s*_*i*__ with standard basis vectors $\tilde{\text{i}}$, $\tilde{\text{j}}$, and $\tilde{\text{k}}$ corresponding to the x-, y-, and z-axis, respectively. Define the origin of the local coordinate as **p**_0_, and


(7) $$\begin{array}{l} \tilde{\text{i}}\equiv \frac{{\text{p}}_{i}-{\text{p}}_{0}}{\left||{\text{p}}_{i}-{\text{p}}_{0}|\right|} \end{array}$$

Then, the rotational direction **r**_*i*_ could be written as


(8) $$\begin{array}{l} {\text{r}}_{i}={\tilde{r}}_{i,1}\tilde{\text{i}}+{\tilde{r}}_{i,2}\tilde{\text{j}}+{\tilde{r}}_{i,3}\tilde{\text{k}} \end{array}$$

where ${\tilde{r}}_{i,1}$ and ${\tilde{r}}_{i,2}$ are equal to 0, and the sign of ${\tilde{r}}_{i,3}$ indicates the direction of possible rotational activities. If the rotation with the centre as **p**_0_ is clockwise, ${\tilde{r}}_{i,3}<0$ and vice versa.

**Remark 1**. $\tilde{\text{i}}$, $\tilde{\text{j}}$, *and* $\tilde{\text{k}}$ *may vary depending on the locations* ***p**_0_, **p**_*i*_*, *and* **p**_*s*_*i*__ for *i* = 1, *…, *n*_*c*_. For spiral catheters (e.g., Lasso, Biosense Webster), ideally all recording points are in the same x-y plane, and* $\tilde{\text{k}}$ *are the same for all* *i* = 1, …, *n*_*c*_. *Thus, the global coordinate and local coordinates could be represented by just one coordinate for simplification. However, the simplification is not applicable for basket catheters*.

With Equations (6) and (8), CIV is calculated as


(9) $$\begin{array}{l} CIV=\frac{\left|\underset{i}{\sum }\text{sign}\left({\tilde{r}}_{i,3}\right)\right|}{{\text{n}}_{n}\left({\text{p}}_{0}\right)} \end{array}$$

where n_*n*_(**p**_0_) is the number of available neighbouring recordings around **p**_0_ to quantify the rotational activity. CIV ranges from 0 to 1 and measures the circulatory propagation patterns, whereby spatially stable RD yield a high CIV and meandering unstable RDs a low value. Details of using CIV for RD detection and its validation can be found in the method section and in our recent study by Handa et al. (2020).

#### 2.2.3. Labelling

In the patient with persistent AF group, each subject underwent detailed intracardiac mapping in the atrium with a 20-electrode AFocusII mapping catheter, (St Jude Medical, MN, USA). Multiple areas were mapped within the atrium with the catheter recording 20 separate EGMs at a time. CPI, a measure of organisation of fibrillation, was calculated for each set of AFocusII recordings in a given region. Global CPI was calculated as the average of the CPI of all the regional AFocusII recordings for the subjects. The criteria for binarising the 17 subjects in the clinical AF data sets is shown in Figure 1B. In particular, those with RD-positive areas and CPI above the median CPI were labelled as organised, and those without any RD-positive areas or CPI below the median CPI as disorganised.

## 3. Method

### 3.1. QRS Subtraction

**Figure 2:**
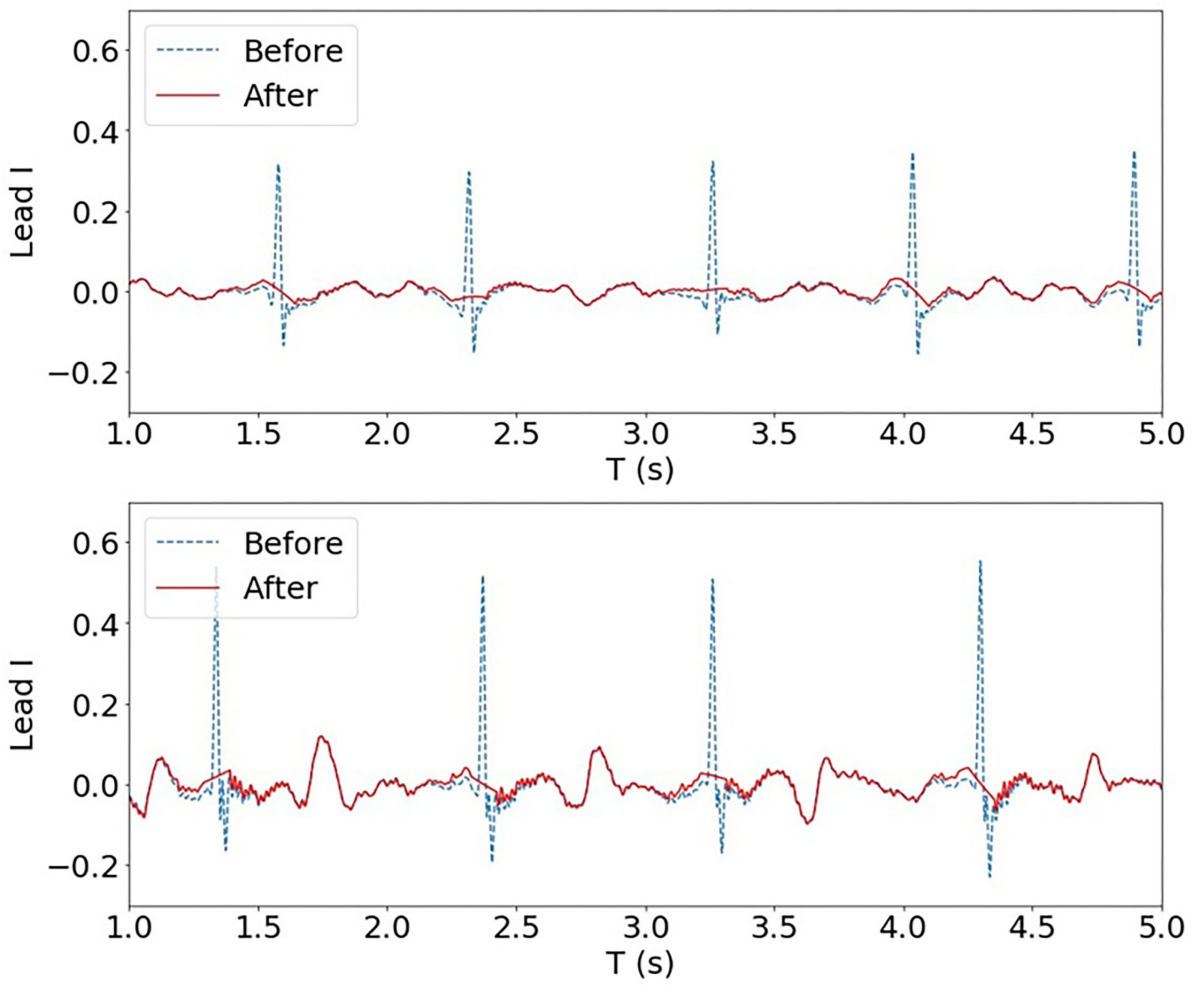
Two examples of QRS subtraction applied to the AF ECG data set (Lead I). Upon QRS detection, points corresponding to a QRS duration of 0.10 s were replaced with linearly interpolated points.

The frequency spectrum of the individual QRS complex is often found in a range of 10~30 Hz (Bollmann et al., 2006), and the frequencies characterising the atrial signal are mostly confined to the interval of 5~12 Hz (Lin, 2008). Due to this overlap between atria electrical activity and QRS complexes, QRS subtraction was applied to the clinical AF ECG data set. In particular, QRS detection followed by linear interpolation proposed in Ahmad et al. (2011) was adopted. Normal QRS duration is between 0.08 and 0.10 s. To ensure ventricular activity fully removed, points corresponding to a QRS duration of 0.10 s were subtracted and replaced with linearly interpolated points with a ratio of 5:6, i.e. with 5/11 points before and 6/11 points after the peak detection, as described in Ahmad et al. (2011). Two examples of QRS subtraction are shown in Figure 2.

### 3.2. Feature Extraction

In this study, we propose to use the band-power (BP) feature, i.e., the power of the ECG signals corresponding to different frequency bands, to classify the organisation type. Given the heart rate in rats is markedly higher than in humans, in this study, different bands and data segmentation settings were selected for rat VF and clinical AF ECG feature extraction.

For the rat VF model, the single-channel continuous ECG recordings were sampled at a sampling rate of 1,000 Hz and segmented by a 2 s window with a window shift of 1 s. The DF of rat VF ranges from 10~20 Hz (Handa et al., 2018). Thus, the segmented data were filtered with eight temporal philters with a bandwidth of 4 Hz ranging from 2~3 4Hz (2~6 Hz, 6~10 Hz, … 30~34 Hz, fourth-order band-pass Butterworth philters with an allowance of 2 Hz). In addition to band-power, an AR model was applied to boost the number of features for the single-channel rat VF ECG. The AR coefficients with the order of 20 together with the BP of the eight bands were concatenated and used as the feature vector. Thus, the total number of features for rat VF ECG was 28.

For the clinical AF dataset, the continuous 12-lead ECG recordings were sampled with a sampling rate of 2034.5 Hz and segmented by an 8 s window with a window shift of 4 s. The segmented data were then filtered into four bands, i.e., 5~15 Hz, 15~25 Hz, 25~50 Hz, and 50~100 Hz (fourth order band-pass Butterworth philters with an allowance of 2 Hz). The DF of AF was found to be within the frequency spectrum of 3~12 Hz. Thus, most of the AF components can be covered by the band 5~15 Hz. The other three higher bands are selected to capture subtle high-frequency characteristics of the signals. For each band and each lead, the band-power was calculated then normalised by the total power of the signal of a broad band 2~200 Hz. With four normalised BP features extracted for each lead, the total number of features for a clinical AF ECG segment was 48.

### 3.3. Feature Selection

Mutual information is a measurement of the dependence between features and class labels and has been successfully applied for feature selection of BP features of time series (Ang et al., 2012). Thus, in this study, mutual information was adopted for feature selection.

Given the feature *f* and class label *c*, the mutual information is formulated as below:


(10) $$\begin{array}{l} I\left(f,c\right)=H\left(c\right)-H\left(c|f\right) \end{array}$$

where *H*(*c*) is the individual entropy of class label *c* and *H*(*c*|*f*) is the conditional entropy of class label *c* given feature vector *f*. It could be interpreted as the amount of uncertainty reduced in the class label *c* through observing feature *f* Ang et al. (2012).

In this study, the class label *c* was the organisation level. For each BP or AR feature, its mutual information with class label *c* was calculated during the training stage. The top 50% of features with the highest mutual information were selected and used in the classification step.

### 3.4. Classification

In this study, binarised linear discriminative analysis (LDA) and a linear regression model were adopted for the organisation level prediction. During the model training and testing, features from 2 s segments of ECG were used. With the prediction results at the segment level, the final organisation level prediction of each subject was obtained by voting or averaging. For each subject, the final class label was the class label that was predicted most frequently during the segment classification. The subject was predicted as organised if more than half of the segments were predicted to be organised. For clinical AF data, a linear regression model was also tested for continuous organisation level prediction. With the regression model, the mean predicted value of all segments was used as the final prediction of the subject.

The whole evaluation ran in LOO manner, whereby the feature selection and classification framework were trained by data segments from eight out of nine subjects (rat VF ECG) or 16 out of 17 subjects (clinical AF ECG) and evaluated on segments from the remaining subject. Thus, for different subjects, different features could have been selected in the training. In this way, the data segments for each subject were not used to train the model that they were tested with.

## 4. Experimental Results

### 4.1. GC Analysis of Clinical AF Data

**Figure 3:**
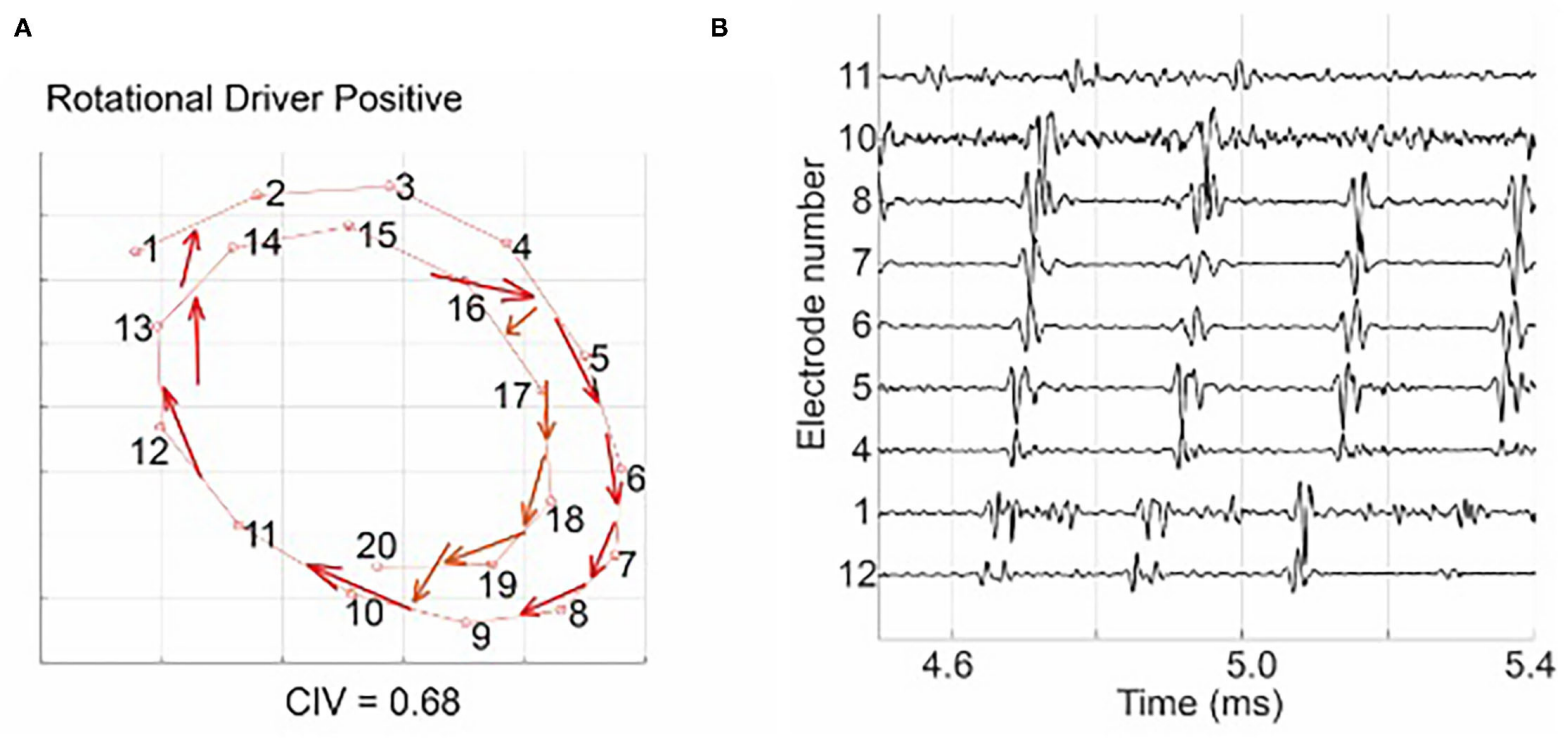
**(A)** shows a source-to-sink vector map constructed from the intra-cardiac EGM signal, indicating a site with a stable RD with a high circular interdependence value (CIV). The arrows show the directions of the source-to-sink vectors. The corresponding EGMs in **(B)** show organised clockwise rotational activation: electrode 12 was activated first, followed by electrodes 1, 4–8, 10, and 11, and the activation of the electrode 11 was followed by the next activation of electrode 12. Figure reproduced from Handa et al. (2020) (CC BY 4.0).

Figure 3A shows an example of a source-to-sink vector map constructed for a set of intracardiac multipolar (AFocusII) catheter recordings in AF. It shows a site with a stable RD with a high CIV driving a globally organised form of AF. The arrows show the directions of the source-to-sink vectors. The corresponding EGMs show organised clockwise rotational activation. For this example, the CIV of 0.68 was above the threshold of 0.60, which is the operating point obtained using rat VF data in our previous study for classifying an RD-positive area (Handa et al., 2020).

**Figure 4:**
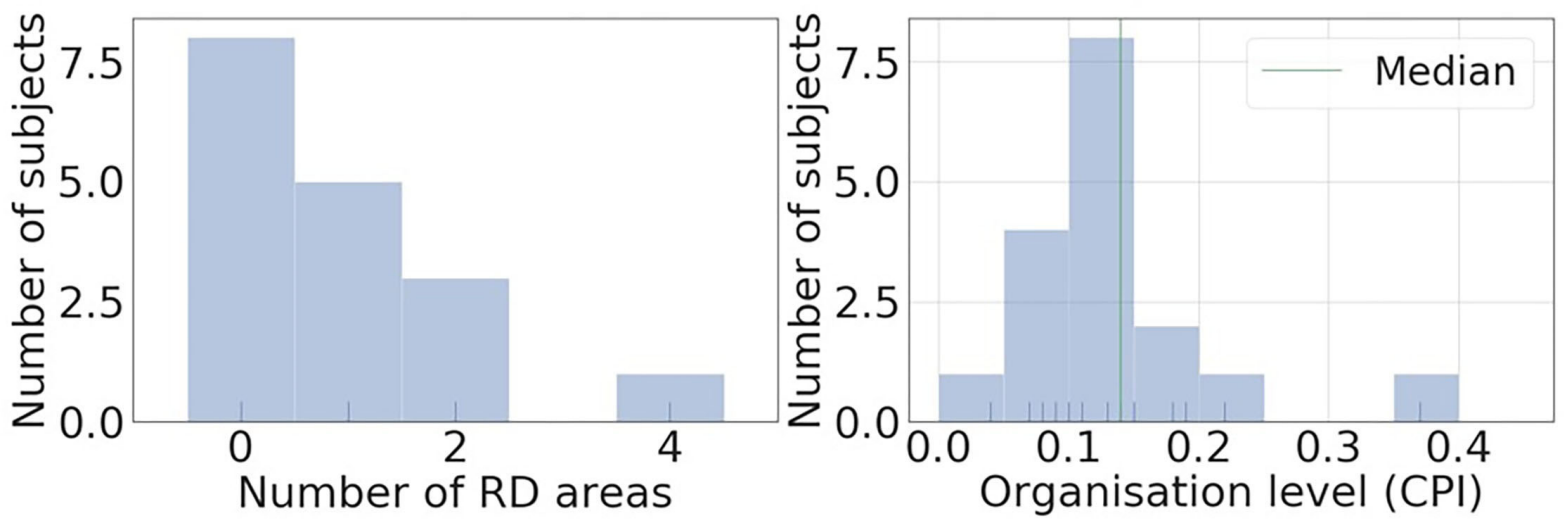
Histograms of RD positive areas and causal pairing index (CPI) of the 17 subjects of clinical AF ECG/EGM dataset. Most of the subjects have less than 2 RD positive areas, and the median CPI of all subjects is 0.14.

Figure 4 shows histograms of RD-positive areas (Figure 4A) and CPI (Figure 4B) of the 17 subjects of the clinical AF ECG/EGM dataset. For this data set, 8 out of 17 subjects have no RD-positive area, and the maximum number of RD-positive areas identified with intracardiac mapping is 4. The median CPI of all subjects is 0.14. Among the nine subjects with RD-positive areas, the five subjects with CPI above 0.14 were labelled as organised, and those without any RD-positive areas or CPI below the median CPI as disorganised.

### 4.2. Statistical Correlation Analysis

To investigate whether the surface ECG BP reflected the AF organisation level measured invasively, Pearson correlation analysis was performed to test the correlation between normalised band-power features and the organisation level as quantified by CPI for the clinical AF ECG. Logarithm was applied to make the BP distribution normal distribution. A *p*-value smaller than 0.05 was considered statistically significant.

**Figure 5:**
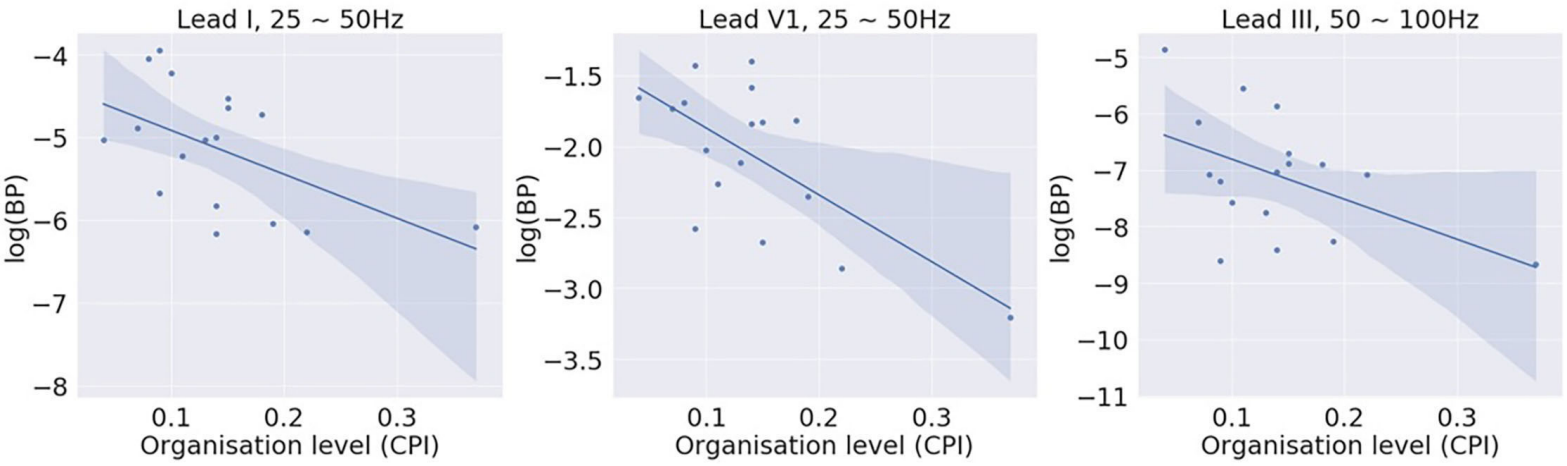
Correlation between log (band power) of different bands and the global fibrillatory organisation level measured with causal pairing index (CPI). Significant correlations were found for higher frequency bands 25~50 Hz and 50~100 Hz with certain leads.

Figure 5 shows examples of the correlation results of leads I, V1, and III, with each closed circle representing one subject. Table 2 summarises all significant correlations with *p* < 0.05. The significant correlations were found in two relatively higher bands, 25~50 Hz and 50~100 Hz. The correlation was the strongest with BP of lead V1, 25~50 Hz (β = –0.67; *p* < 0.01). No significant correlation was found with bands of lower frequencies, i.e., 5~15 Hz and 15~25 Hz. The correlation analysis demonstrates that there is a negative correlation between the amount of high-frequency components in the signal and the level of global fibrillatory organisation for certain leads.

**Table 2:** Correlation results with clinical atrial fibrillation (AF) data.

**Band**	**Lead**	**β**
25~50 Hz	I	–0.53^*^
25~50 Hz	aVL	–0.56^*^
25~50 Hz	V1	–0.67^**^
50~100 Hz	III	–0.49^*^
50~100 Hz	aVL	–0.59^*^

* *α = 0.05,*

** *α = 0.01*.

### 4.3. Organisation Level Classification of ECG

**Figure 6:**
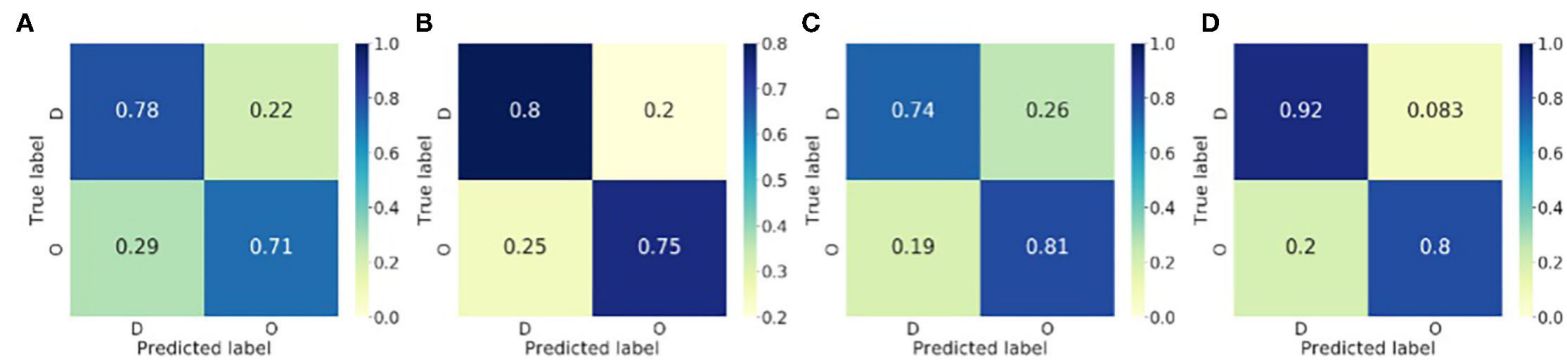
Confusion matrices of the binary classification of the fibrillatory organisation level of the rat VF and clinical AF data. **(A)** Rat VF ECG, segment level. **(B)** Rat VF ECG, Subject level. **(C)** Clinical AF ECG, segment level. **(D)** Clinical AF ECG, subject level.

The proposed method was evaluated by LOO, and subsequently, the numbers for the training and test data passed to the classifier varied. For the rat VF classification, the number of training samples ranges from 1,856 to 2,416, and that of the test from 17 to 577. For the clinical AF data, the number of training samples ranges from 1,368 to 1,491, and that of the test from 37 to 160. The LOO classification results for both rat VF and clinical AF data are summarised in Table 3, where *c* and ĉ are the true and predicted class labels, respectively, and ‘**O**' and ‘**D**' represent the organised and disorganised classes, respectively. *n*_*seg*_ is the total number of the data segments per subject, and for the clinical AF data, the number of areas being mapped *n*_*k*_ was also included. For each subject, the final prediction was calculated by voting: the subject would be organised if more than 50% of the segments were predicted as organised, and disorganised if below (or equal to) 50%. In Table 3, *P*_**w**_ is also presented, which is the percentage of segments classified as the winner class during voting. The confusion matrices of the classification at segment and subject levels were shown in Figure 6. For the subject-level prediction, the sensitivities are 75 and 80%, the specificities are 80 and 91.67% and the accuracies are 77.78 and 88.24% for rat VF and clinical AF, respectively.

**Table 3:** Leave-one-out (LOO) classification results (%).

**Subject ID**	***n*_*seg*_ (*n*_*k*_)**	**c**	**ĉ**	***P*_w_ (%)**
Rat-1	168	O	D	76.78
Rat-2	196	O	**O**	73.98
Rat-3	439	O	**O**	98.61
Rat-4	577	O	**O**	100.54
Rat-5	162	D	**D**	71.43
Rat-6	179	D	**D**	77.09
Rat-7	17	D	O	100.00
Rat-8	434	D	**D**	82.10
Rat-9	261	D	**D**	93.87
Clinical-1	160 (21)	D	**D**	63.12
Clinical-2	47 (8)	D	**D**	100.00
Clinical-3	44 (9)	D	**D**	100.00
Clinical-4	85 (16)	D	**D**	100.00
Clinical-5	148 (28)	D	**D**	68.24
Clinical-6	76 (15)	D	**D**	59.21
Clinical-7	53 (9)	D	**D**	100.00
Clinical-8	131 (20)	D	**D**	87.79
Clinical-9	97 (16)	D	O	91.75
Clinical-10	67 (9)	O	D	100.00
Clinical-11	123 (25)	O	**O**	98.37
Clinical-12	151 (27)	O	**O**	97.35
Clinical-13	80 (10)	D	**D**	61.25
Clinical-14	40 (6)	D	**D**	87.50
Clinical-15	91 (25)	O	**O**	98.90
Clinical-16	98 (15)	D	**D**	100.00
Clinical-17	37 (6)	O	**O**	54.05

*Bold values indicate correct predictions by the machine learning algorithm. i.e. c = ĉ*.

**Figure 7:**
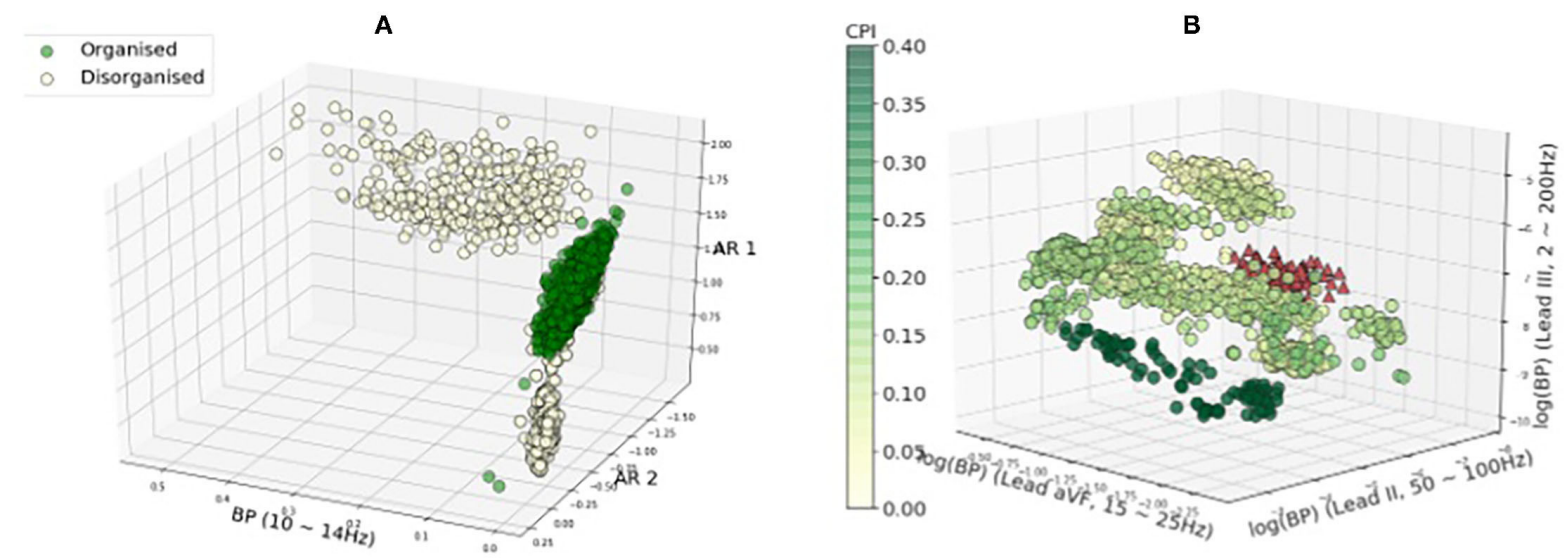
Feature distribution regarding the fibrillatory organisational level. The three best features selected by mutual information were plotted. The colour of the features are corresponding to the CPI values except features of subject clinical-10 in red. In General, the features with the high and low CPI are separated. However, features of subject clinical-10 with high CPI (in red triangles) tend to be overlapped with features from the low-CPI group. **(A)** Rat VF features. **(B)** Clinical AF features.

Figures 7A,B show the distribution of the three most discriminative features selected using mutual information for rat VF ECG and clinical AF ECG, respectively. In both panels, each circle represents one feature calculated from a 2 s data segment. Note that the feature selection was only applied for illustrative purposes. For the rat VF ECG in Figure 7A, most segments from the organised class fell within one cluster, separated from the two clusters of disorganised features. For Rat-7, the duration of VF is shorter, resulting in a much smaller number of available segments than the other rat subjects. Thus, the short ECG segment may not be able to fully represent the fibrillatory characteristic, which could be the reason for its incorrect classification with high *P*_**w**_ = 100%.

Figure 7B shows the features of the clinical AF ECG, which formed more sub-clusters. The colour of the features are corresponding to the CPI values except for features of subject clinical-10 in red, as clinical-10 is the only organised subject that is classified as disorganised. Generally, the features with the highest and lowest CPI tended to be separated from each other, while the features with intermediate CPI tended to have more overlapping. Clinical-10 has a CPI of 0.22 and stable RD identified, however, the features of this subject tend to be closer to features from the disorganised class, which could be the reason for the wrong classification. Clinical-9 has a CPI of 0.15 and was labelled as disorganised because no RD is detected for this subject. The reason for the subject being classified wrongly could be the CPI is very close to the median CPI that is used to binarise the data into two groups.

**Figure 8:**
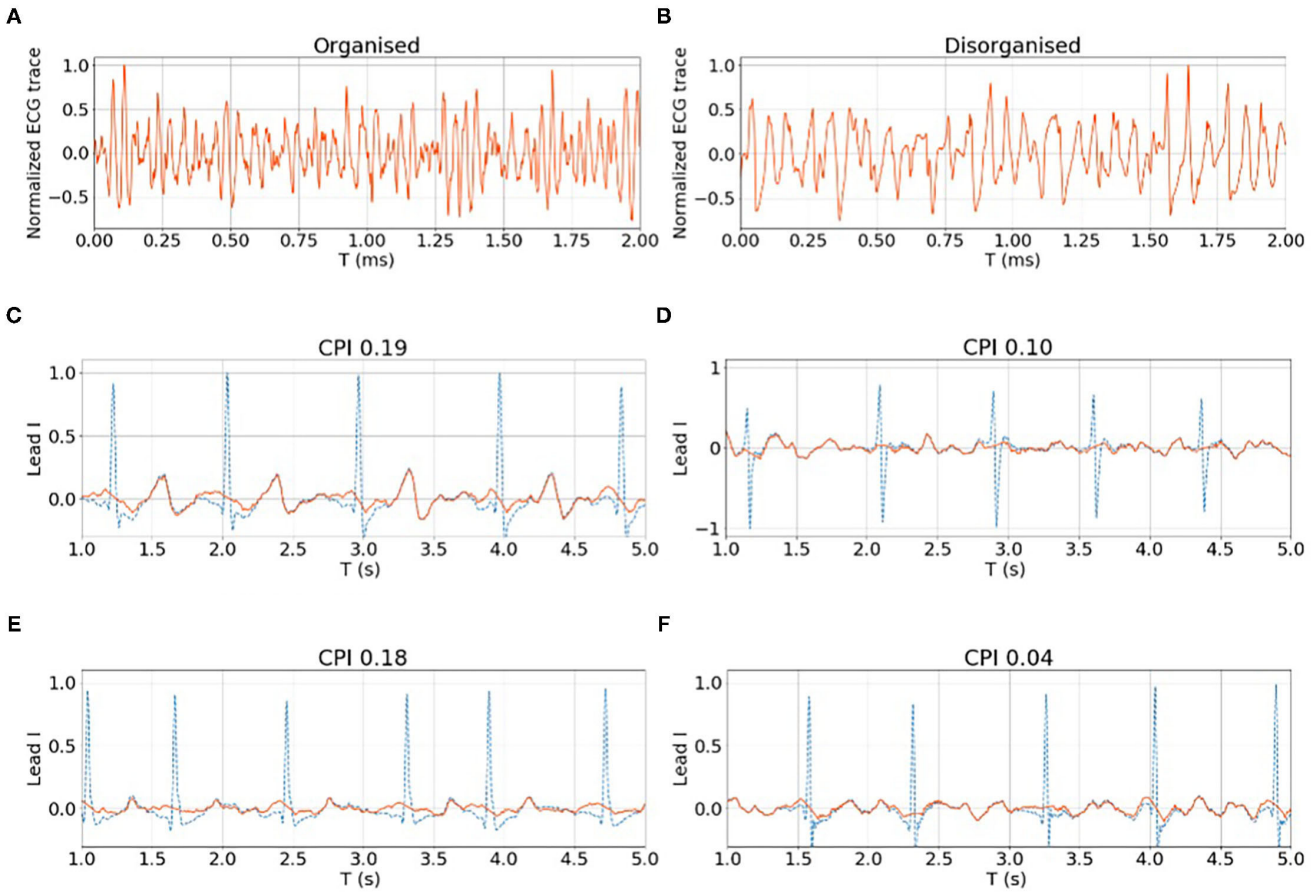
**(A,B)** show examples of organised and disorganised rat VF ECG. **(C–F)** shows examples of clinical AF ECG with different CPI levels calculated from the corresponding EGM data. For both rat and clinical data, it is difficult to differentiate the organised and disorganised data by visual evaluation alone.

Figures 8A,B show examples of the rat VF ECG segments from organised and disorganised classes based on the phase mapping. In Figures 8C–F, examples of clinical AF ECG segments of lead I are shown with the corresponding CPI. Generally, there are no clear morphological patterns associated with CPI level and subsequently, it is difficult to discern the organisation level of a given data segment by visual evaluation alone.

## 5. Discussion

Experimental results showed that the proposed classification methods can differentiate fibrillation of different organisation levels using the surface ECG an with accuracy of 78% for the rat VF data and an accuracy of 88% for the clinical AF data. Based on the results, this method has the potential to non-invasively the determine degree of organisation to aid mechanism-directed treatment decisions for patients with AF and in VF survivors.

The concept of ‘organisation' within AF is not fully established or defined, in part due to a limited understanding of the underlying mechanisms. The degree of complexity within AF has been analysed previously by both local and multi-site analysis of EGMs in time and frequency domains (Ravelli and Masè, 2014). Some groups have looked at analysing the repetitive nature of wavefronts in AF using techniques such as similarity index (Ravelli et al., 2005) and Retro-Mapping (Mann et al., 2019). These techniques require intracardiac electrogram analysis from invasive mapping. Lankveld et al. (2014) previously showed that the spatiotemporal organisation of the AF ECG as measured by techniques such as F-wave complexity, harmonic decay, and DF analysis could delineate paroxysmal AF from the more disorganised persistent AF (Lankveld et al., 2014). Furthermore, it was shown that these AF complexity parameters derived from surface ECGs could predict procedural outcomes from catheter ablation in patients with persistent AF at long-term follow-up (Lankveld et al., 2016). The proposed methodology in this study for characterising the complexity of fibrillation from ECGs has the strength of being both non-invasive and being validated with detailed optical mapping studies in rat VF. The binary classification of AF ECGs as organised or disorganised with regard to description of the probable underlying mechanism may be useful in selecting appropriate treatment strategies for patients. Patients with disorganised AF are likely better suited for treatment with anti-arrhythmic drugs or extensive compartmentalisation of the atria with a surgical approach, while those with an organised AF may benefit from catheter ablation.

### 5.1. Implication of High-Frequency Components

This study shows that the power of frequency bands of relatively higher frequency is negatively correlated with the organisation level, which is consistent with existing studies that organised AF tends to have a lower DF (Lankveld et al., 2016).

**Figure 9:**
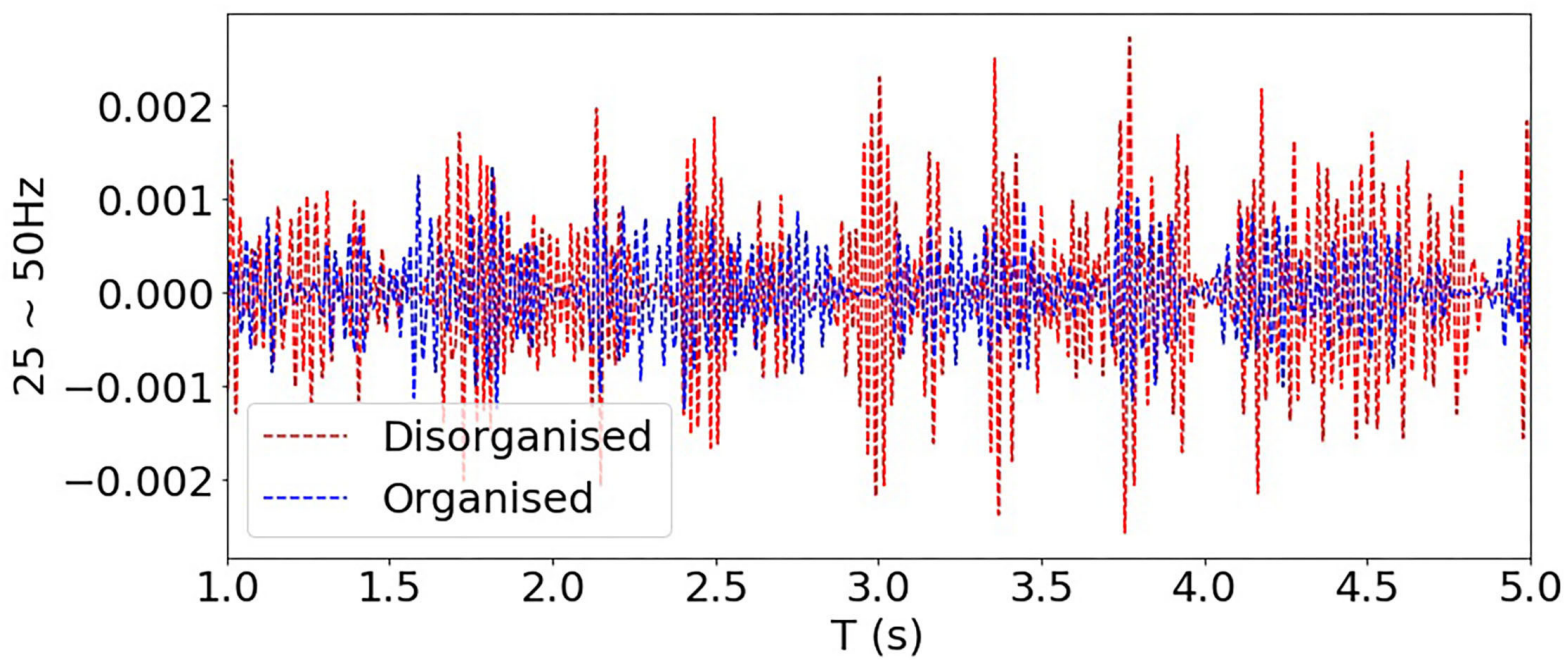
Comparing filtered signals of bands 25~50 Hz from one organised and one disorganised subjects (clinical AF dataset).

In Figure 9, filtered signals (normalised by the total power) corresponding to bands 25~50 Hz (i.e., high-frequency components) from one organised and one disorganised subject were compared, and it shows that the power of these high-frequency components from the disorganised subject was consistently higher than that from the organised subject, although high-frequency components (>25 Hz) constitute a relatively small part of the whole signal spectrum, they may be helpful in distinguishing organised from disorganised AF.

Optical mapping data and EGM recordings can be regarded as sources, and the surface ECG can be regarded as a linear mixture of the sources due to the volume conduction effect. The organised fibrillation driven by one or two stable RDs tends to have activation with consistent and synchronised patterns of activation, yielding the power spectral density (PSD) of the ECG concentrating on a few low-frequency components. When the fibrillation is chaotic with randomly propagating wave-fronts, source signals tend to be more fragmented, resulting in the surface ECG with more high-frequency components.

It is also worth noting that the temporal morphological characteristics of ECG signals are only one aspect of the differences implied by high-frequency bandpower features between the organised and disorganised classes. The significant associations between high frequency bandpower and organisation level were found with only certain leads. Only using features with significant correlations yielded classification accuracies around the chance level. Moreover, using features from all 12 leads but from only one single band, 25~50 Hz or 50~100 Hz, also yielded accuracies around 60%. Neither a single band nor a single lead can fully capture the source pattern differences between the organised and disorganised subjects. The different spatial dispersion patterns over the 12 leads of different frequency bands are the key in discriminating the organised and disorganised classes.

### 5.2. Lead Optimisation

For the 12-lead clinical ECG, we also estimated the lead weights by solving the following optimisation problem


(11) $$\begin{array}{l} \hat{\text{w}}=\arg\underset{\text{w}}{\min}\frac{\text{𝔼}\left[{\left({\text{w}}^{⊤}{\text{x}}_{o}\left(t\right)\right)}^{2}\right]}{\text{𝔼}\left[{\left({\text{w}}^{⊤}{\text{x}}_{d}\left(t\right)\right)}^{2}\right]} \end{array}$$

where $\text{w}\in R^{12}$ is the channel weights, and *x*_*o*_(*t*) and *x*_*d*_(*t*) are the vectors of the band-passed ECG signal at time *t* of organised and disorganised classes, respectively. ${\text{w}}^{⊤}{\text{x}}_{o}\left(t\right)$(${\text{w}}^{⊤}{\text{x}}_{d}\left(t\right)$) can be regarded as a single virtual channel, and $E\left[{\left({\text{w}}^{⊤}{\text{x}}_{o}\left(t\right)\right)}^{2}\right]$ and $E\left[{\left({\text{w}}^{⊤}{\text{x}}_{d}\left(t\right)\right)}^{2}\right]$ denotes the expectation of the BP of the single virtual channel for organised and disorganised classes, respectively. By solving (Equation 11), the lead weights could be optimised in a way that **w** maximises the difference between the BP of the organised and disorganised classes. We have applied (Equation 11) to each band and used the bandpower of the single virtual channel as the feature. This approach was evaluated in the same LOO manner. However, the lead optimisation based on Equation (11) is not as good as that using mutual information for feature selection, possibly due to over-fitting. Selecting the leads yielding BP features with the highest mutual information means that weights of leads could be either 0 or 1. This process involves fewer parameters to be tuned as solving (Equation 11), and subsequently, is more robust against the cross-subjects dissimilarities within the same class.

### 5.3. Limitations

A limitation of this study is that the AF mapping data were low-resolution sequentially acquired intracardiac EGM data used to label the underlying mechanism, and follow-up data were not available. Thus, the ground truth for the organisation level could not be directly determined and had to be inferred from GC analysis. GC analysis of intracardiac electrogram in patients with AF was established from a methodology developed from analysis of rat VF optical mapping. The outcomes measured in AF in this study may have been influenced by mapping resolution, interelectrode distance, catheter stability, and heterogeneity in mapping. CPI value to determine the fibrillatory organisation, while applied to an unselected population may also have been influenced by the characteristics of this population.

Because the sample size of the clinical AF data is small, it is difficult to infer the true distribution of the CPI of clinical AF. In this study, we combined the RD detection results with median CPI to binarise the data. A more comprehensive data set, e.g., including cardiac imaging data from patients and follow-up data post ablation would be needed for a better binarisation in future study. Furthermore, in this study we have conducted QRS subtraction while it is difficult to fully remove T-waves while keeping the fibrillation signals intact. In our future study, we will seek better signal processing approaches for fibrillation signal extraction.

## 6. Conclusion

Individualised mechanism–directed treatments with better patient selection are needed for myocardial fibrillation treatment. If the mechanism of myocardial fibrillation, specifically AF, can be determined from the surface ECG, patients can be non-invasively screened for specific treatment strategies, whereby only patients with globally organised fibrillation are candidates for targeted ablation of drivers, and those with globally disorganised fibrillation are better treated with anti-arrhythmic drugs or ablation strategies to compartmentalise the atria. In this study, we propose a classification framework for detection of the fibrillation organisation level, and thus, the underlying fibrillation mechanism (stable RD vs. multiple wavelet driven) from the ECG alone, with no need for invasive intracardiac recordings.

Experimental results in this study showed that the proposed classification methods can differentiate fibrillation of different organisation types: for the rat VF ECG, the sensitivity, specificity, and accuracy are 75, 80, and 78%, respectively; and when these methodologies were adapted for the clinical AF ECG, the sensitivity, specific, and accuracy are 80, 92, and 88%, respectively. Therefore, the proposed techniques in this study have the potential to determine fibrillatory mechanisms and may aid non-invasive mechanism-directed tailoring of treatments for patients with AF and in VF survivors.

## Data Availability Statement

The raw data supporting the conclusions of this article will be made available by the authors, without undue reservation.

## Ethics Statement

The studies involving human participants were reviewed and approved by Local Research Ethics Committee (Bromley). The patients/participants provided their written informed consent to participate in this study. The animal study was reviewed and approved by Imperial College London Ethical Review Board.

## Author Contributions

XL and FN: conception. XL, BH, NQ, ZW, NL, PL, PK, NP, and FN: data collection. XL, BH, and BZ: data analysis. XL, XS, BH, AS, and FN: drafting article. All authors: critical revision of the article and final approval.

## Funding

This work was supported by the British Heart Foundation (RG/16/3/32175) and the National Institute of Health Research (NIHR), Imperial Biomedical Research Centre.

## Conflict of Interest

FN, BH, XL, and NP are inventors on a patent application on Granger Causality mapping. The remaining authors declare that the research was conducted in the absence of any commercial or financial relationships that could be construed as a potential conflict of interest.

## Publisher's Note

All claims expressed in this article are solely those of the authors and do not necessarily represent those of their affiliated organizations, or those of the publisher, the editors and the reviewers. Any product that may be evaluated in this article, or claim that may be made by its manufacturer, is not guaranteed or endorsed by the publisher.

## Figures and Tables

**Figure A1:**
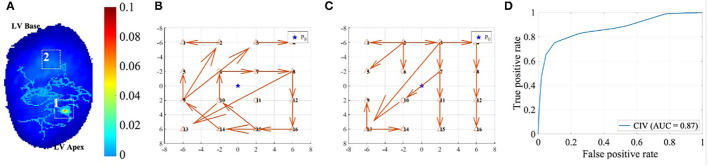
**(A)** shows the down-sampling of the high-resolution optical mapping data of rat VF. Areas 1 and 2 were labelled according to the RD (RD) heatmap shown as the background, the local GC vector maps of which were shown in **(B)** and **(C)**, respectively. **(D)** shows the ROC of the RD prediction using CIV.

## A. Appendix

Circular interdependence value for RD identification was validated with optical mapping data of the rat VF model. Figure A1 shows an example of the down-sampling optical mapping data. In (Figure 6A), The background is the ground truth of the rotational activity obtained by phase mapping in the form of a heat map, the value of which is the percentage of the time rotational activities staying at the given location. In particular, the red hotspot is of a value around 10%, which is around 350 ms given that the total duration of this recording is 4 s. The DF of the rat VF data usually ranges from 20~30 Hz. Thus, the rotation activity at the red hotspot would have approximately 18 times of rotations, making it a relatively very stable RD site. The original optical mapping data were down-sampled as 4-by-4 grids with 1/4 of the original resolution. Each rat heart would generate approximately 75 down-sampled areas.

Two examples of local GC map after down-sampling are shown in (Figures 6B,C), and areas 1 and 2 were labelled as RD and non-RD class, respectively, according to the heat map in (Figure 6A). The CIV for area 1 is much higher at 0.83 than that of area 2 at 0.27. The RD prediction for all the down-sampled areas using CIV yielded the receiver operating characteristic (ROC) curve shown in (Figure 6D), with an area under curve (AUC) of 0.87 and the best operating point at 0.60.
